# Patient satisfaction with continuous epidural analgesia after major surgical procedures at a Swedish University hospital

**DOI:** 10.1371/journal.pone.0235636

**Published:** 2020-07-02

**Authors:** Egidijus Semenas, Michael Hultström

**Affiliations:** 1 Department of Surgical Sciences, Unit for Anaesthesiology and Intensive Care, Uppsala University, Uppsala, Sweden; 2 Department of Medical Cell Biology, Unit for Integrative Physiology, Uppsala University, Uppsala, Sweden; Cleveland Clinic, UNITED STATES

## Abstract

**Objective:**

The use of epidural analgesia after major surgery is a well-established analgesia method. Epidural analgesia for postoperative pain relief needs to be monitored regularly in order to evaluate patient satisfaction and avoid side effects. However, due to the new available regional techniques, the role of epidural analgesia is being questioned and data about patient satisfaction is lacking. The current study was designed to evaluate patient satisfaction with epidural analgesia, its efficacy and reasons for premature termination of epidural analgesia.

**Materials and methods:**

We conducted a retrospective survey of all patients who undergone surgery at Uppsala University hospital between October 2012 and January 2014 requiring continuous epidural analgesia for postoperative pain relief. Patients’ satisfaction with epidural analgesia and its effectiveness were evaluated by using paper questionnaire.

**Results:**

During the study period 579 epidurals were inserted in patients scheduled for vascular, hepatobiliary, esophageal and other major abdominal surgery. The average treatment time was 3.8±1.8 days. Epidural analgesia consisted either of bupivacaine 0.1%+sufentanil 1 μg/ml solution or ropivacaine 0.2% solution. If patient needed opiates during treatment with epidural analgesia, only ropivacaine 0,2% solution was used. 494 (87.9%) patients were satisfied with their analgesia with no difference in satisfaction between sexes being observed. In 62 cases (11.2%) patient controlled analgesia was used on top of epidural analgesia with ropivacaine 0.2% solution, and 50.8% of patients were satisfied in this group. 514 (91.4%) patients were reported as having a good effect, 24 (4.3%) patients reported or were tested to show some effect, and 24 (4.3%) had no effect. No major neurological complications (epidural hematoma or abscess) were observed.

**Conclusions:**

Our retrospective survey indicates that patients are satisfied with continuous epidural analgesia used in major surgery.

## Introduction

Epidural analgesia has been successfully used for postoperative pain relief for many years. There is enough evidence that epidural analgesia is superior to a patient controlled opioid-based therapy [1, 2]. Although epidural analgesia is well established for provision of good analgesia following a major abdominal surgery [3], however, due to the new available regional techniques, the role of epidural analgesia is being questioned [4].

In contrast to the subjective experience of many anesthetists, failure of epidural anesthesia and analgesia is a frequent clinical problem. In a cohort of 2140 surgical patients, failure rates of 32% for thoracic and 27% for lumbar epidural were described [5, 6]. In addition, delivery of effective epidural analgesia is far from guaranteed.

The current retrospective study was designed to evaluate patient satisfaction with epidural analgesia and its efficacy. The hypothesis was that patients with continuous epidural analgesia experience good pain relief as well as high satisfaction. Finally, the reasons for premature termination of epidural analgesia at the ward were also evaluated.

## Materials and methods

The study was approved by Uppsala ethics committee (Dnr 2014/194, 2014-05-28). The study was a single-centre retrospective study. The need for informed consent was waived by the ethics committee.

All patients scheduled for a major surgery between October 2012 and January 2014 and requiring epidural analgesia for postoperative pain relief were included in the study.

Patients scheduled for either an elective or emergency higher (proximal to the ligament of Treitz) or lower abdominal surgery (distal to the ligament of Treitz), urologic surgery, vascular and orthopedic surgery were included in the survey. The attending anesthetist decided if epidural analgesia was needed and chose medications for epidural infusion accordingly. The epidural catheter was inserted at the level most appropriate for the type of the surgery.

Prior to the induction of anesthesia, an epidural catheter (Portex Epidural Minipack System 2; Smiths Medical, St. Paul, Minnesota, USA) was placed using a standard 18-gauge Tuohy needle. During the surgery a commercially produced solution of bupivacaine 0.1%+sufentanil 1 μg/ml (BS) (APL Pharma Specials, Stockholm Sweden) or ropivacaine 0.2% (R) ((APL Pharma Specials, Stockholm Sweden) was given via a pump (CADD-Legacy PCA Pump Model 6300; Smiths Medical, St. Paul, Minnesota, USA). The intra- operative infusion rate or a temporary discontinuation of the epidural infusion due to a cardiovascular instability was left to the discretion of the attending anesthetist.

After the surgery, the patient was transferred to the postoperative care unit (PACU). The background infusion rate was titrated by the attending PACU anesthetist within the range of 3 to 10 ml/h (for bupivacaine 0.1%+sufentanil 1 μg/ml solution) or up to 12 ml/h (for ropivacaine 0.2% solution). The aim was to achieve a numerical rating scale (NRS) pain level of less than 4 without causing hypotension (a systolic blood pressure lower than 90 mmHg) or other side effects. All patients included in the study had an active epidural infusion at the time of discharge from PACU. If epidural analgesia was unsatisfactory or the patient was taking opioids, patient controlled intravenous analgesia (PCA) was activated. PCA was only administered on top of epidural analgesia. If it was decided to add PCA, an epidurally administered solution was always changed to ropivacaine 0.2%. The policy was never to administer a strong opioid intravenously together with the epidurally administered sufentanil. The usual dose of PCA drug was 2mg (morphine 5mg/ml or ketobemidone 5mg/ml). The lock-out time was 15 minutes.

All discharge criteria had to be fulfilled before the transfer from PACU to a general surgical ward. These included a circulatory and respiratory stability, an awake and alert state, ability to communicate, NRS less than 4 and no motor block.

An acute pain service consisting of one anesthesia nurse made rounds on the surgical wards every day. The anesthetist on-call was responsible for answering questions and solving acute problems related to epidural analgesia. During the rounds a special chart was used where variables of interest were noted down. The following data was recorded: the level of insertion of the epidural catheter; the number of treatment days, the infusion rate and the total amount of boluses administered to each patient, epidural solution, the reason for the termination of treatment, satisfaction level (satisfied, moderately satisfied, unsatisfied) at termination of the treatment, efficacy (effective, moderately effective, ineffective), intravenously administered patient controlled analgesia (PCA, morphine 5mg/ml or ketobemidone 5mg/ml, decided by responsible anesthetist) and other additional painkillers. Bolus doses requested and given were recorded on a daily basis by the ward staff. Paracetamol was given routinely throughout the postoperative period, whereas NSAIDs or opioids were given as indicated.

### Statistical analysis

Data is expressed as mean ± standard deviation or as median and interquartile range (IQR). Frequencies are expressed as a number of cases (percentage). Differences in frequency were analysed using Chi-square. A logistic general linear model was used to compare the effects of factors believed to affect the chance of successful epidural analgesia.

## Results

During the study period 579 epidurals were inserted. 17 patients were excluded from the final analysis: data was missing for 4 patients, 8 epidurals were inserted before the surgery but were not activated afterwards, and 5 epidurals were inserted during the surgery by the surgeon. Thus, 562 epidurals were used in the final analysis.

### Effectiveness

514 (91.4%) patients were reported as experiencing a good effect, 24 (4.3%) patients reported or were tested to show some effect, and 24 (4.3%) patients had no effect (see S1 Appendix).

### Satisfaction

Satisfaction in patients was primarily analysed based on the distinction between satisfied (1) vs not satisfied (2 or 3) (see S1 Appendix). 494 (87.9%) patients were satisfied with their analgesia. Patients who started with BS and did not change were satisfied in 91.4% of cases, patients who were started on R were satisfied in 81.0% of cases, and patients who changed from BS to R were satisfied in 62.5% of cases. In 62 cases (11.2%) patient controlled analgesia (PCA) was used on top of epidural analgesia with R, and 50.8% of patients were satisfied in this group. There was no difference in satisfaction between the sexes (female = 86.3% compared to male = 88.9% [Chi^2^ = 0.63 and p = 0.42]). Data about patients’ satisfaction and factors influencing it is presented in Tables 1 and 2, respectively.

**Table 1:** Epidural data and patients’ satisfaction with epidural analgesia after different types of surgery.

	High abdominal surgery	Low abdominal surgery	Vascular surgery	Urological surgery	Orthopedic surgery
Level of insertion	Th8 (Th7-Th9)	Th10 (Th9-Th11)	Th11 (Th10-L1)	Th10 (Th9-Th11)	L1 (Th12-L3)
Total number of patients (n = 562)	n = 204	n = 172	n = 40	n = 82	n = 64
Satisfaction					
Percentage of satisfied patients	81.4	93.0	81.3	95.1	95.0

Levels of insertion are shown as median (IQR).

“n” means the number of patients. Mean age was 64 years with a range between 18 and 95, median 67 with IQR 57–74. 40.4% of patients were women and 59.6%—men.

**Table 2:** Influence of different factors on patients´ satisfaction with epidural analgesia (logistic modelling).

Type of surgery and number of patients		95% confidence interval		
	Effect (RR, relative risk)	2.5%	97.5%	P =
High abdominal surgery (n = 204)	1			
Low abdominal surgery (n = 172)	3.0	1.5	6.4	0.002 **
Orthopedic surgery (n = 64).	1.8	0.8	4.4	NS
Urological surgery (n = 82)	3.8	1.4	13.1	0.02 *
Vascular surgery (n = 40),	3.3	0.9	20.9	NS
B+S	1			
R	0.4	0.2	0.9	0.03 *
Change to R	0.2	0.1	0.5	P <0.001 ***
Age	1.03	1.01	1.05	0.002 **

B+S, bupivacaine 0.1%+sufentanil 1 μg/ml solution. R, ropivacaine 0.2% solution.

NS = not significant.

### Choice of local anesthetic

483 (85.9%) of patients started with BS and 79 (14.1%) started with R. 40 patients (7.1%) who started with BS changed to R at a later stage.

### Reasons for premature discontinuation

A total of 23 epidurals were discontinued prematurely after surgery. Causes for premature termination of epidural analgesia are presented in Table 3.

**Table 3:** Causes for premature termination of epidural analgesia at the ward.

Cause of premature termination	Number of patients (%)
Dislodgement	9 (1.5%)
Leakage	6 (1.0%)
Motor block	3 (0.5%)
Hypotension (systolic blood pressure <90 mmHg)	1 (0.2%)
Pruritus	2 (0.4%)
Stroke	1 (0.2%)
Sedation	1 (0.2%)

### Duration of treatment

The average treatment time was 3.8±1.8 days (median 4, IQR 2–5).

## Discussion

This survey of continuous epidural analgesia has confirmed that most patients are satisfied with their postoperative analgesia at this centre. There is no accepted or universal standard for evaluating patient satisfaction after postoperative epidural analgesia [7]. Patients were asked about their overall satisfaction at the termination of epidural treatment. We found that a survey posing a single question *Are you satisfied with your postoperative analgesia*? and three possible answers -*satisfied*, *unsatisfied or moderately satisfied*—is easy to perform as well as to understand. Even if answers like *unsatisfied or moderately satisfied* are considered as not satisfactory, the results of satisfaction (87.9%) in the study are comparable to other studies (87%) [7]. More patients were satisfied if epidural analgesia was started with BS compared to R (91.4% vs 81%). This is an expected outcome since R is the first choice for patients where the EDA is not expected to be enough, or the change from BS to R is only done if additional opiates are needed. It is interesting to note that the number of treatment days or the patient’s sex were not associated with patients´ satisfaction. However, a surgery type, choice of the drug and age were associated with the satisfaction level, with patients operated for lower abdominal or urological procedures being more satisfied (93% and 95.1%, respectively) compared with patients who underwent other higher abdominal procedures (81.4%). This decrease in satisfaction may be related to the extensive nature of the surgery, including the liver, the pancreas and oesophageal resections [8]. Besides, it is more technically challenging to put high thoracic epidurals compared to lower thoracic epidurals. Better pain control reducing incisional pain likely reduces respiratory splinting and encourages early ambulation that may reduce atelectasis and the development of post- operative pneumonia [9]. Thus we may speculate that patients after lower abdominal or urological surgery are easier to ambulate compared to extensive upper abdominal procedures. This improvement in ambulation may also influence patients´ overall satisfaction. In our study an orthopedic surgery proved to be a factor causing lower satisfaction. Orthopedic patients are in more pain before procedures and are usually taking different types of painkillers, including opiates. Thus, an epidural catheter is only a part of the multimodal pain treatment plan. Higher age was also associated with better satisfaction with an effect of about 3% per year-of-age possibly driven by uniformly high satisfaction in the highest age-group and more varied in the younger group.

Most patients who were satisfied with analgesia also had an effective epidural (91.5%). The epidural solution used in our institution (local anaesthetic together with opiate) is well known and safe [10, 11]. The overall failure rates between 7.5 and 25% have been reported in other studies [6–7, 12–13]. In our study, dislodgement of the catheter and leakage from the puncture site were the most common grounds to terminate the treatment (2.5%). The observed incidence is much lower compared to other studies [6, 14]. We may speculate that the daily care performed by ward nurses, guided by an acute pain care nurse, helped to decrease significantly the failure rate observed in other similar studies. Our results confirm that if epidural analgesia started during the surgery is sub-optimal, an active management of epidural treatment (a new catheter insertion, change of the epidural solution due to side effects and PCA activation at PACU) results in an almost complete success rate [13]. Pruritus is a very common and usually a mild side effect of opioid-based epidural analgesia [15]. However, in our study 2 patients (0.35%) had to discontinue their treatment due to pruritus compared to 5 patients out of of 4135 in Golster et al. study (0.12%) [7]. We used the epidural solution containing sufentanil 1 microgram/ml compared to fentanyl 18 microgram/ml used by Golster et al. [7]. 3 patients had to change to the ropivacaine 0,2% infusion due to pruritus caused by the bupivacaine-sufentanil solution. In our study, only in 22 (3.77%) cases epidural analgesia was terminated prematurely.

A relatively low number of patients and a lack of long-term follow-up with regard to potential neurological complications are the main limitations of our study. However, no cases of neurological complications were observed during the study itself.

## Conclusion

The study confirmed that patients were satisfied with continuous epidural analgesia used in major surgery at the Swedish University hospital.

## Supporting information

S1 Appendix (DOC)
