# Supplementary material for: Patient satisfaction with continuous epidural analgesia after major surgical procedures at a Swedish University hospital
Source: PLoS One. 2020 Jul 2;15(7):e0235636. doi: 10.1371/journal.pone.0235636 (PMC7331990; doi:10.1371/journal.pone.0235636)
Supplement: S1 Appendix — (DOC) [file pone.0235636.s001.doc]

# Evaluation form for continuous epidural treatment

An acute pain service consisting of one anesthesia nurse made rounds on the surgical wards every day. The anesthetist on-call was responsible for answering questions and solving acute problems related to epidural analgesia (EDA). During the rounds a special chart was used where variables of interest were written. The criteria used to evaluate the effectiveness of continuous epidural treatment at the surgical ward are presented below. EDA effectiveness was evaluated taking into account the surgical region it was supposed to cover. Continuous EDA treatment was combined with paracetamol. A breakthrough pain episode which needs treatment– the pain according to VAS/NRS scale > 3 at rest or > 5 at movement.

VAS= Visual analoge scale

NRS= Numeric rating scale

## Criteria for evaluation of the effectiveness of EDA treatment:

## Good effect

EDA treatment is effective.

1. According to VAS/NRS scale, the patient´s pain is ≤ 3 at rest and ≤ 5 at movement.
2. A satisfactory effect of EDA boluses, i.e. when a bolus dose is given/administered, the patient feels that the pain decreases to ≤ 3 at rest or ≤ 5 at movement.
3. At postoperative care unit – an EDA catheter is withdrawn or the level of paresthesia/motor weakness is deemed to be acceptable for EDA treatment continuation at the surgical ward.

## Moderate effect

At least one of the following:

1. A patient experiences > 4 episodes of the breakthrough pain per 24 hours as of the second postoperative day, on several days, despite the increase of the epidural infusion rate to the maximum or the change of epidurally administered drugs (from bupivacaine-sufentanil to ropivacaine).
2. Not satisfactory effect of EDA boluses, i.e. the patient feels that the pain does not decrease to ≤ 3 at rest or ≤ 5 at movement or to the level the patient thinks is satisfactory.
3. EDA treatment has to be optimized at the recovery unit after the patient returned to the surgical ward.

## Bad effect

At least one of the following:

1. EDA treatment is discontinued due inadequate analgesia.
2. EDA treatment is discontinued due to paresthesia, motor weakness or other serious side effects.
3. Need to choose another pain treatment method (any regional blockade).
4. Need to put a new epidural catheter (does not include cases where epidural catheter glides out).

#### Patient’s satisfaction with epidural analgesia

The patient is asked the following question at the end of the treatment envolving an epidural catheter: *Are you satisfied with your postoperative analgesia?*

Three possible answers:

##### Yes (= satisfied, 1) Moderately satisfied (2) No (= unsatisfied, 3)
